# Tackling Photonic Inverse Design with Machine Learning

**DOI:** 10.1002/advs.202002923

**Published:** 2021-01-07

**Authors:** Zhaocheng Liu, Dayu Zhu, Lakshmi Raju, Wenshan Cai

**Affiliations:** ^1^ School of Electrical and Computer Engineering Georgia Institute of Technology Atlanta GA 30332 USA; ^2^ School of Materials Science and Engineering Georgia Institute of Technology Atlanta GA 30332 USA

**Keywords:** inverse design, machine learning, nanophotonics, neural networks

## Abstract

Machine learning, as a study of algorithms that automate prediction and decision‐making based on complex data, has become one of the most effective tools in the study of artificial intelligence. In recent years, scientific communities have been gradually merging data‐driven approaches with research, enabling dramatic progress in revealing underlying mechanisms, predicting essential properties, and discovering unconventional phenomena. It is becoming an indispensable tool in the fields of, for instance, quantum physics, organic chemistry, and medical imaging. Very recently, machine learning has been adopted in the research of photonics and optics as an alternative approach to address the inverse design problem. In this report, the fast advances of machine‐learning‐enabled photonic design strategies in the past few years are summarized. In particular, deep learning methods, a subset of machine learning algorithms, dealing with intractable high degrees‐of‐freedom structure design are focused upon.

## Graphical abstract

Employing engineered photonic structures for unconventional control of light represents a central theme in modern optics. The last few years have witnessed a growing trend of exploring artificial intelligence for the design of photonic materials and devices. Herein, the fast advances of machine‐learning‐enabled photonic design strategies are summarized, with particular emphasis on deep learning methods that tackle high degrees‐of‐freedom structures.

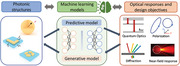

## 1 Overview

Over the past two or three decades, the exploration of artificially structured photonic media has represented a central theme in the optical sciences. By carefully engineering photonic structures to be comparable with or smaller than the wavelength, light behaviors, and properties like transmittance, polarization, chirality, and frequency, can be accurately manipulated in unprecedented manners. As such, artificial photonic structures are enabling tremendous applications in modern optical engineering and advanced science research, such as virtual/augmented reality,^[^ ^1^ ^]^ sensing technologies,^[^ ^2^ ^]^ optical system miniaturization,^[^ ^3^ ^]^ and optical communications.^[^ ^4^ ^]^ Nowadays, research in photonics has branched out to various fields with substantial influence in the scientific community. For example, photonic crystals^[^ ^5^ ^]^ consist of repeating regions of distinct refractive indices, enabling allowed and forbidden spectral ranges of light and controlling the propagation of light inside the crystal. Plasmonics^[^ ^6^ ^]^ studies how light gives rise to and interacts with collective excitations of free electrons at metal surfaces, manipulating light waves down to the deep subwavelength scale. By introducing spatial variations in the optical response of miniature light scatterers, metasurfaces^[^ ^7^, ^8^ ^]^ enable arbitrary wavefront shaping with unprecedented flexibility by producing controllable abrupt changes in the phase, amplitude, and polarization of light waves. Apart from the enumerated cases, there are several specific disciplines of photonics, and the unique characteristics of artificial structures of photonic devices offer the possibility for extensive applications.

Analogous to the subject of macroscopic artificial structures, the design of microscopic structures remains a major topic in photonic research. Although photonic structure performance is typically straightforward to predict, through sophisticated simulation algorithms such as finite element method (FEM) and finite different time domain (FDTD), the inverse problem, designing an on‐demand photonic device, is not closed‐form. At the early stages of nanophotonics research, the prototypical designs were mostly based on educable guesses such as the split‐ring,^[^ ^9^ ^]^ V‐shaped antenna,^[^ ^8^, ^10^ ^]^ and gammadions^[^ ^11^ ^]^ to name a few. However, limited by the prior knowledge of humans and the complicated light‐matter interaction mechanisms, photonic devices with unconventional functionalities and extremely high efficiencies may have never been discovered with intuitively guessed geometries. In order to address the difficulty of photonic and optical design, inverse design methodologies, such as adjoint methods^[^ ^12^ ^]^ and evolutionary algorithms,^[^ ^13^ ^]^ have become one of the main themes of photonics research in recent years. These algorithms have successfully been implemented for the design of various unconventional photonic devices, such as power splitters,^[^ ^14^ ^]^ light trapping structures,^[^ ^15^ ^]^ and dielectric nanoantennas.^[^ ^16^ ^]^ In order to further expand the capabilities of machine‐aided design approaches, and to avoid some downsides of traditional optimization (such as the local minimum problem and expensive computations), the optical community has started to look at data‐driven and machine learning methods as alternative approaches to address the inverse design problem.

In the past two decades, the prevalence of information technology and the advances of hardware have been greatly accelerating machine learning and data science development. As such, machine learning has become the central research theme in computer vision, natural language processing, speech recognition, and much more. Besides commercial and engineering applications, machine learning is assuming an ever‐growing importance in scientific research. For example, it is becoming an indispensable tool for the design of molecule structures,^[^ ^17^, ^18^, ^19^ ^]^ planning of chemical syntheses,^[^ ^19^, ^20^ ^]^ prediction of material functionalities,^[^ ^18^, ^21^ ^]^ classification of celestial bodies,^[^ ^22^ ^]^ detection of high energy particles,^[^ ^23^ ^]^ and investigation of many‐body systems.^[^ ^24^ ^]^ Recently, the optical community has been progressively migrating the techniques of machine learning and data science into photonics research, with a number of successful applications including ultrafast optics,^[^ ^25^ ^]^ optical communication,^[^ ^26^ ^]^ and optical microscopy.^[^ ^27^ ^]^ Machine learning has also helped control the active meta‐atoms for microwave applications and realize self‐adaptive invisible metasurface cloak in response to incident wave.^[^ ^28^ ^]^ On the other hand, researchers are seeking methods from photonics to solve machine learning problems. An exciting interdisciplinary example is the development of optical analog modules to accelerate the mathematical operations in deep neural networks by leveraging nanophotonic circuits^[^ ^29^ ^]^ and diffractive optics.^[^ ^30^ ^]^ The fast processing speed enabled by light‐matter interaction at the wavelength scale is pushing photonic chips as a competitive candidate for the next generation of processing units serving large‐scale deep learning inference.

With the astronomical capability of capturing essential features from vast amounts of high‐dimensional data, machine learning models have become a promising tool to aid photonic design in various ways. In this report, we will introduce the fast advances in machine learning techniques and their applications to the design and optimization of photonic structures. Specifically, we focus on the design approaches relying on deep learning models that tackle high degree‐of‐freedom (DOF) designs. In the following discussion, we will first cover the basics of deep learning, with a glimpse of the general deep learning methods in photonic inverse design. Then, several deep‐learning‐enabled design methods with their applications in various photonic design tasks are introduced. In the last section, we will include several design strategies with a consolidation of both machine learning algorithms and traditional optimizations. In some cases, such a hybrid strategy shows a prominently enhanced design capability over the solely data‐driven methods or traditional optimization algorithms.

## 2 Brief Introduction to Deep Learning

In this section, we will provide a general introduction to deep learning fundamentals. We will list a few essential deep learning architectures and their applications without diving into details, and then have an overall discussion of discriminative and generative models. In the last part of this section, we will illustrate the general methodology of implementation for deep learning models in the inverse design of photonic devices.

### 2.1 Categories of Deep Learning Architectures

Modern deep learning architectures are based on neural networks, which are inspired by the learning patterns in biological nervous systems. Generally speaking, a neural network is composed of multiple layers of artificial neurons, each performing a certain transformation on its input information. With the combined transformations throughout all layers, the neural network is essentially capable of representing arbitrary real‐valued functions. In optical and photonics research, three different architectures are commonly mentioned: the fully connected network (FCN), the convolutional neural network (CNN), and the recurrent neural network (RNN).

The FCN is the primitive type of neural networks. An FCN consists of multiple layers of neurons, and each neuron is connected to all the neurons on the adjacent layers. The fully connected properties provide the FCN sufficient capacity to mimic any complicated transformations. However, the dense connection also consumes large computing resources. To relax the computational cost without sacrificing the performance of neural networks, CNN is coined as an improved alternative. Instead of calculating the weights between all the connected neurons, each layer of a CNN conducts cross‐correlation operations between the incoming tensor and the convolutional kernel. Such operations maintain the translate invariance over the input tensor, enhancing the efficacy of capturing features from image/audio data with strong spatial/temporal correlations. When it comes to sequential data, RNNs are the paradigm that is mostly used. RNNs can be explicitly generalized as sequentially connected neurons. An RNN is able to process the input data at different timestamps one at a time and generate a sequence of data based on the input time series.

### 2.2 Discriminative and Generative Model

Machine learning models can be roughly categorized into two classes: discriminative models and generative models. Mathematically, a discriminative model predicts the probability of label *y* conditioned on the input data *x*, i.e., *p*(*y*|*x*), while the generative learns a probability distribution of *x, p*(*x*) or a joint probability of *x* and *y*, *p*(*x*, *y*). Intuitively, a discriminative model is a function *f*(*x*), transforming the input data into a label or value *y = f*(*x*). Thus, discriminative models are always related to classification and regression tasks in supervised learning schemes. A generative model, on the other hand, can map the data *x* into a compact representation, and with some sample algorithm, we can retrieve more data that is similar to the input dataset. Generative models usually serve in the unsupervised learning paradigms, such as classification without labels. It should be noted that the introduction to generative and discriminative models is not rigorous or complete, and there are different interpretations of the two models.

Regarding the implementation of the two models, if we use deep neural networks, we can say the models are deep discriminative/generative models. In specific, deep discriminative models are typically a direct implementation of fundamental network architectures such as an FCN or CNN. With sufficient training data, which could be parameters of the photonic structures, and labels, which could be spectral responses, we can have the modern deep learning frameworks take care of the training of the network. However, the implementation of generative models is not as straightforward as discriminative models, as generative models are built upon several interactive network modules. The essential usage of deep generative models in photonic research is to either capture the distribution of datasets to provide insights of design, or to perform dimensionality reduction to simplify the optimization. In a latter section, we will have a very brief introduction of generative models that have been used in photonics research with concrete examples.

### 2.3 Incorporating Machine Learning into the Design of Photonic Devices

**Figure 1:**
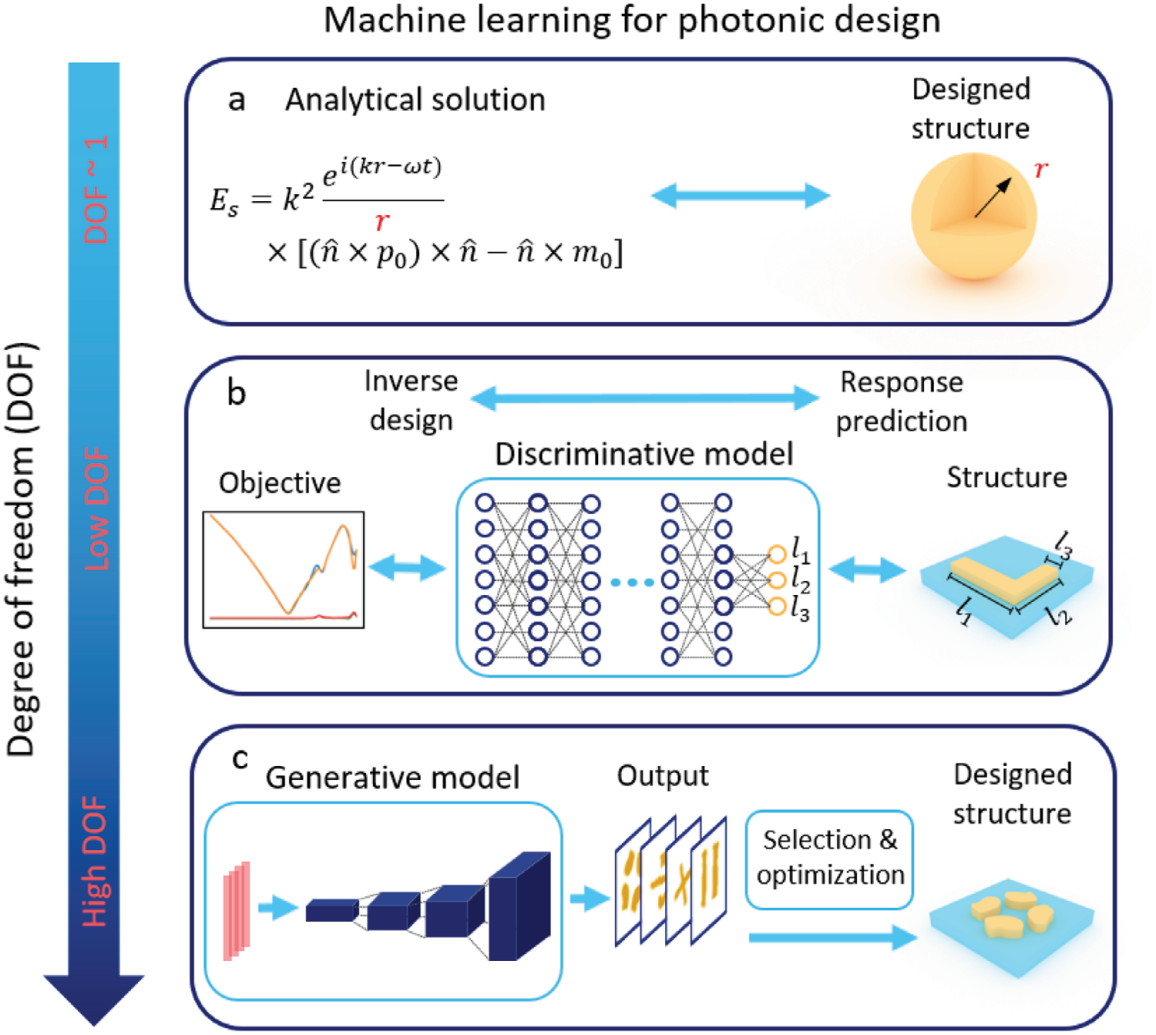
Methodologies of photonic design through machine learning at different degrees of freedoms (DOFs). a) When the DOF of the photonic structure is low, the optimal combination of the design parameters can be found by analytical solution or simple parametric sweeping. However, such a design strategy may not yield optimal performance. b) As the DOF of the design grows, the solution space expands. Without a proper optimization algorithm, the identification of optimal designs requires exponentially increasing iterations of simulation when the dimensionality of the design space grows. In the context of machine learning, we may use discriminative model to capture the relations between design parameters and optical responses with substantially reduced amount of data. It should be noted that, since multiple configurations of structures may correspond to the same response, a single discriminative model is not able to perfectly map an optical response back to a unique set of design parameters. Additional training strategies are required if discriminative models are used for the optimization and design. c) When the DOF continues growing to thousands and more, generative models can help to reduce the dimensionality of the design and to seek relations between design parameters and optical responses for further optimization. The generative models can be jointly leveraged with discriminative models as well as traditional optimization algorithms to speed up the design process or to locate the global optimal solutions.

The machine learning methodologies used in photonic inverse design are associated with the DOF of the photonic structures. We have listed the general implementation of machine learning models in various design tasks in terms of the DOF as shown in **Figure** **1**. Traditionally, with only a few parameters to optimize as in Figure 1a, analytical calculation and parametric sweeping are sufficient. With an increased DOF, the optimization space grows fast and simple parametric sweeping cannot yield a satisfactory result. In order to accelerate the optimization process and explore new designs, discriminative models have been proposed to assist the design as shown in Figure 1b. Typically, discriminative models are used to learn the bidirectional mapping of the optical structures and their optical responses. However, since multiple distinct optical structures may correspond to an identical optical response (which is also called the degeneracy problem), the mapping from optical responses back to the structural parameters requires additional processing. We will list several proposed methods that can avoid the degeneracy problem in the next section.

When the DOF continues growing to thousands and more, the immense dimensionality of the optimization space invalidates the approaches that require huge amounts of data or vast iterations of simulations. Generative models, in this case, can be leveraged to reduce the dimensionality of the design structures and optical responses. Two of the most fundamental and widely used deep generative models are generative adversarial networks (GANs)^[^ ^31^ ^]^ and variational autoencoders (VAEs).^[^ ^32^, ^33^ ^]^ Although the architectures, performance, and training approaches of GANs and VAEs differ, both GANs and VAEs are able to capture the distribution of high‐dimensional data and represent them in a reduced dimensional space, which is also called a latent space. By sampling the latent space, generative models can produce more data that are “similar” to the training dataset. As shown in Figure 1c, a trained generative model is able to encode the photonic structure data into a compact representation, and to produce unlimited photonic structures by feeding reduced dimensional vectors to the model. Optimal design can be exhaustively searched from the compact representation. Besides, because the generative model is essentially transforming the original data to another representation, the local minima in the original parameter space are also varied and possibly eliminated in the transformed one. This property may alleviate the local minimum problem if optimization is performed in the sparse representation. Another usage of the generative model is to encode high‐dimensional data into a compact representation, to provide insights on the relations among data to assist the optimization. As we will see in the later section, generative models can also be incorporated to the traditional optimization algorithms to assist fast design and avoid local minimum problem.

## 3 Discriminative Models

In this section, we will introduce several successful implementations of discriminative models in the design of photonic structures and devices. The many‐to‐one mapping from the structural space to the response space makes the inversion non‐unique; the design cannot be optimally achieved through simply training a single discriminative model. Hence, auxiliary training strategies and optimization schema are required to assist the design process. Several exemplary strategies that avoid such a degeneracy problem will be discussed. In addition, we will also present some outstanding works for accurately predicting optical responses of photonic structures with a deep learning model. Such a deep‐learning‐based method enables fast evaluation of the design performance during the computationally intensive optimization.

### 3.1 Design of Metallic Metasurfaces and Metamaterials

**Figure 2:**
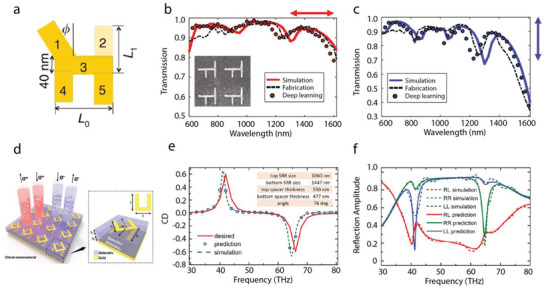
Design of metallic metasurfaces and metamaterials for amplitude and chirality manipulation. a–c) Design of “H” shaped metallic metasurfaces. a) Schematic of the shape and parameters of the nanostructure. b,c) The simulated, measured, and deep learning retrieved spectra of the design when the incident light is horizontal and vertical polarized, respectively. The SEM image of the fabricated sample is shown in the inset of b,d–f) Design of chiral metamaterials. d) Schematic of the designed chiral metamaterial. The inset is the zoomed‐in structure of a single meta‐atom. e) Desired, predicted, and simulated circular dichroism (CD) spectra. The insets list the retrieved geometric parameters. f) Predicted full reflection spectra along with the full‐wave simulation results. a–c) Reproduced under the terms of the Creative Commons CC‐BY license.^[^ ^34^ ^]^ d–f) Reproduced with permission.^[^ ^36^ ^]^ Copyright 2018, Americal Chemical Society.

Malkiel et al. first proposed solving the inverse design of nanostructures in metasurfaces defined by a few parameters through a bidirectional neural network.^[^ ^34^ ^]^ The particle with an “H” shape in the demonstration is illustrated in **Figure** **2**a. Two networks, a geometry‐predicting‐network (GPN) and a spectrum‐predicting‐network (SPN), are built to collectively solve the design problem. The GPN predicts the geometric parameters given a spectral response, while the SPN approximates the spectrum of the input geometry of a structure. During the training process, the output of the GPN is fed into the SPN. Given a training pair composed of geometric parameters and its corresponding spectrum, the aim is to minimize the loss between the training pair and the outputs of the GPN and SPN. After the training of the bidirectional network, new designs with various desired responses can be generated expeditiously by feeding the objectives into the GPN. Figure 2b,c shows an example of a retrieved structure given two spectral behaviors with *x*‐ and *y*‐ polarized incident light. The SEM of a fabricated nanostructure with a thickness of 40 nm is shown in the left corner of Figure 2b. The network‐predicted, FEM simulated, and measured results of nanostructure are presented by solid, dashed, and circled lines, respectively. Similar strategies leveraging two networks, one of which is for approximating optical responses and the other one for predicting design parameters, have also been shown to be powerful in other design applications such as broadband highly reflective metasurfaces.^[^ ^35^ ^]^

Another bidirectional strategy was developed and extended to the design of multilayered chiral metamaterials as illustrated in Figure 2d.^[^ ^36^ ^]^ The unit cell of the chiral metamaterials is composed of two stacked gold split ring resonators (SRRs). The chirality of such a metamaterial is characterized by circular dichroism (CD), which is defined as the difference of the absorption of left and right circularly polarized incident lights. The overall network architecture for the design is constructed through two bidirectional networks—a primary network and an auxiliary network. The primary network learns the mapping between the optical spectra and the design space, while the auxiliary directly tackles the relation between the CD and its corresponding structure parameters. The incorporation of the auxiliary network further improves the accuracy of the prediction and design. An example of the retrieval of the structure and its optical responses is shown in Figure 2e,f. Given a desired CD shown as a red line in Figure 2e, the network generates a structure with the simulated CD (dashed lines) matching the desired one. Figure 2f also provides the simulated spectrum of the designed structure.

### 3.2 Design of Layered Photonic Structures

**Figure 3:**
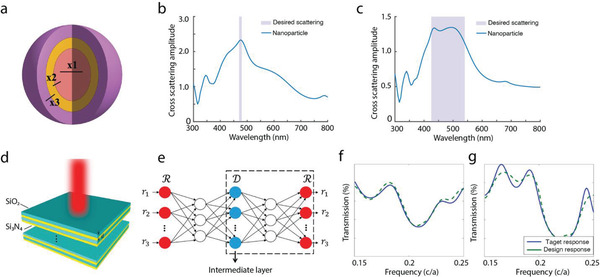
Design of layered photonic structures. a–c) Design of spherical nanoparticles. a) The schematic of the target core–shell nanoparticle. The design objective is to maximize the cross‐scattering amplitude given a range of wavelengths. b,c) Examples of the on‐demand design results, where the simulated cross‐scattering amplitude of the optimized structures are presented, given narrow‐ and broad‐band design requirements, respectively. d–g) Thin film photonic structure design. d) Schematic of the design configuration, which is composed of 16 layers of SiO_2_ and Si_3_N_4_ thin films. The design objective is to optimize the thickness of each layer given the desired transmission spectrum. e) Illustration of the design network architecture, which is a combination of an inverse design network and a forward modeling network. f,g) Examples of the design results, where the target responses and the responses of the designs are displayed as solid and dashed lines, respectively. a–c) Reproduced with permission.^[^ ^38^ ^]^Copyright 2018, The Authors. d–g) Reproduced with permission. ^[^ ^39^ ^]^ Copyright 2018, American Chemical Society.

Besides the metasurface design, deep learning‐based design approaches have also been applied to the design of layered photonic structures and particles in the application of photonic crystals, scattering manipulation, and analog computing.^[^ ^37^ ^]^ Peurifoy et al. proposed a strategy for the design of multilayer dielectric spherical nanoparticles.^[^ ^38^ ^]^ The design particle has a silica core, and alternating TiO_2_ and silica shells as shown in **Figure** **3**a. The optimization of design parameters is realized by the backpropagation algorithm enabled through the automatic differentiation of the deep learning frameworks. In detail, a forward network is pretrained with the simulated dataset, and then the weights of the network are fixed. The design parameters are optimized through the iterative backpropagation from the loss function. Figure 3b,c shows two designs of five‐layer particles for the maximum scattering across narrow and broad wavelength ranges, respectively. The backpropagation is essentially a generic gradient descent algorithm, which can locate a local minimum after certain iterations. With the help of deep learning frameworks, the parallel and fast evaluation of the gradients promotes the optimization speed by several orders of magnitudes. This unique feature of deep learning models makes it an increasingly beneficial design method when multiple designs with same configurations are required at once.

Figure 3d–g shows an another example of designing a layered thin film photonic device with a deep learning algorithm.^[^ ^39^ ^]^ The overall device is composed of 16 alternative SiO_2_ and Si_3_N_4_ thin films, as shown in Figure 3d. The objective of the design is to optimize the thickness of each layer to achieve certain target transmission spectra. Same as the inverse design of metasurfaces, different configurations of the structure may correspond to the same optical responses. The authors proposed a two‐step strategy with a design network and a tandem network as shown in Figure 3e to resolve this problem. The tandem network is pretrained to predict the optical responses with an input of design parameters. The design network, with the input of optical responses and output of design parameters, is then trained to reduce the cost function defined as the error between the predicted response and the target response. In doing so, the network overcomes the non‐uniqueness in the inverse design without manually removing the structures with the same responses from the dataset. It is worth mentioning that this training process is similar to the previous example of updating design parameters through backpropagation. However, in this case, the parameters of the design are generated by a network conditioned on the desired spectral behavior, other than directly defined as vectors. Figure 3f,g shows two test examples where the target responses are shown in solid lines and the transmissions of designed films are show in dashed ones. Similar applications of such a method are also proposed in the design of dielectric metasurfaces for color generation ^[^ ^40^ ^]^ and phase/amplitude manipulation.^[^ ^41^ ^]^

### 3.3 Design of High DOF Photonic Device

**Figure 4:**
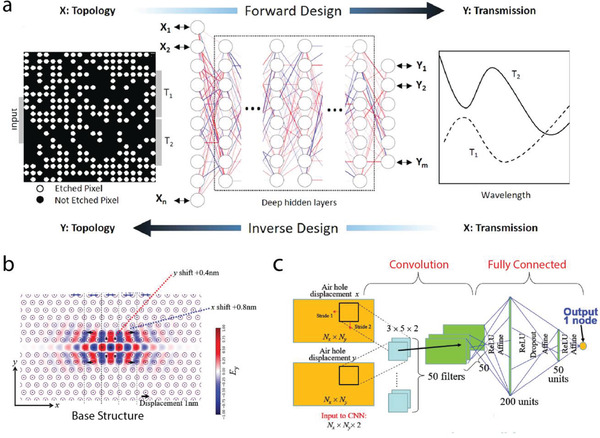
Discriminative models for the design of high DOF photonic devices. a) The design strategy and the network architecture for the 1 × 2 integrated photonic power splitters with various target splitting ratios. The power splitters are represented by a collection of holes to be etched. The forward deep neural network approximates the spectral response at the ports, while the inverse network predicts if the holes should be etched given a desired splitting ratio. b,c) Optimization of 2D photonic crystal nanocavity with CNNs. b) Illustration of the photonic crystal configuration. Circles indicate air holes formed in Si slab. c) Configuration of the neural network that is able to capture the relationship between the displacements of air holes and the *Q*‐factors of the photonic crystal. The optimization is performed by treating the hole displacements as variables and iteratively optimize them through backpropagation. a) Reproduced under the terms of the CC‐BY license.^[^ ^42^ ^]^ b–c) Reproduced with permission.^[^ ^47^ ^]^ Copyright 2018, The Authors.

With the capability of performing regression and classification of high‐dimensional data points, deep neural networks are also leveraged for the design of photonic systems with high DOF. **Figure** **4**a presents a demonstration of a neural network enabled design of silicon‐on‐insulator‐based 1 × 2 integrated photonic power splitters with various target splitting ratios. The power splitters are a collection of holes to be etched represented by pixels in binary images as in the left illustration in Figure 4a. Two networks are also implemented in a bidirectional manner. One of the network models is designed for the forward simulation, i.e., predicting the spectral responses at the output ports given a structure, and the other one is for backward design, i.e., identifying the optimal power splitter with the desired splitting ratio. Since each hole of the splitter can only be etched or not without an intermediate state, the inverse design process is modeled as a classification problem. Skipped connections are incorporated to the network to solve the vanishing gradient problem while training the deep neural networks.^[^ ^42^ ^]^

With the increasing DOF of the design problems, CNNs with shared weights performing the cross‐correlation operation are adopted to efficiently process high‐dimensional data. A CNN is able to capture the local correlation of spatial information in images. As such, a CNN is an ideal candidate to process photonic patterns represented in images, and spectral responses of a given photonic device. CNNs have been utilized in various optical problems, such as the inverse scattering problem,^[^ ^43^ ^]^ wavefront correction,^[^ ^44^ ^]^ digital coding metasurfaces,^[^ ^45^ ^]^ and the prediction of optical properties in complex photonic and materials systems.^[^ ^46^ ^]^ Here we will look at a few examples of CNNs implemented in the design of photonic devices.

Asano and Noda reported a work that utilizes a neural network consisting of CNNs to approximate the *Q*‐factor of photonic crystals and optimize the *Q*‐factor through backpropagation.^[^ ^47^ ^]^ Figure 4b presents an example of a heterostructure 2D photonic crystal nanocavity in the design problem. The aim of the optimization is to identify the positions of these air cavities so as to maximize the *Q*‐factors given a certain initial structure. The network architecture is shown in Figure 4c, where a fully connected network is concatenated after a CNN to predict the *Q*‐factor of the input structure. After the training of the network, the gradient of the cavities’ positions with respect to the *Q*‐factor can be calculated through the backpropagation. Optimization is achieved by iteratively subtracting the gradients from the cavities’ positions. As a demonstration, the optimization strategy dramatically improves the *Q*‐factor of a photonic crystal from 3.8 × 10^8^ to 1.58 × 10^9^. We want to note that CNNs, relying on their capability of processing large dimensional data, have become an indispensable architecture that deal with photonic devices with the high DOF.^[^ ^48^, ^49^ ^]^

### 3.4 Efficient Modeling of Photonic System

When leveraging a discriminative model for the design and optimization of photonic devices, training a surrogate model that approximates the physical responses of the devices is always necessary. For example, deep learning techniques have been leveraged for the modeling of photonic crystals,^[^ ^49^, ^50^ ^]^ metasurfaces,^[^ ^51^ ^]^ and plasmonics.^[^ ^52^ ^]^ The accuracy of the surrogate model determines the fidelity of the design and thus the additional efforts of post‐processing the design. Several strategies can be used for improving the accuracy of the network, and the straightforward way is to augment the dataset by carrying out more simulations. However, additional data is not always a valid solution given limited computing resources and expensive simulations.

**Figure 5:**
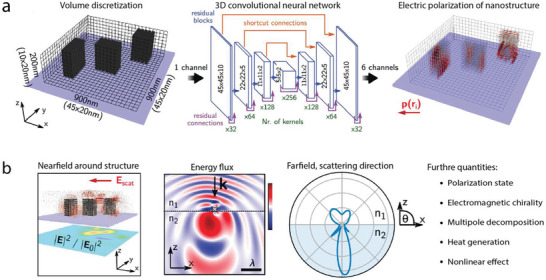
Accurate modeling of multiple optical responses with a single training process. a) Sketch of the neural network model for the modeling of complex optical near‐ and far‐field effects of nano‐optical structure. The photonic structure is represented in 3D grid, and the 3D U‐net is trained to accurately predict the electric polarization density inside of the nanostructure. With the electric information of the structure, all the near‐ and far‐field responses can be computed through various numerical methods without expensive full wave simulations. b) Example quantities that can be derived from the output of the network. Reproduced with permission.^[^ ^54^ ^]^ Copyright 2019, Americal Chemical Society.

To enhance the accuracy of the approximation, various network architectures are explored. For example, residual networks and recurrent neural network are consolidated to accurately predict the optical spectra of input nanostructures represented in images.^[^ ^50^ ^]^ In addition, we want to highlight the work utilizing the U‐Net^[^ ^53^ ^]^ to capture a range of complex optical near‐ and far‐field effects of nano‐optical structures with a single training procedure.^[^ ^54^ ^]^ Such a strategy is able to improve the prediction accuracy while extensively reducing the amount of training data for various design tasks. The architecture and algorithm of the framework is illustrated in **Figure** **5**a. A nanostructure with an arbitrary shape is mapped onto the 3D grid, and the neural network model is trained to predict the electric polarization density inside the nanostructure with training data simulated by coupled dipole approximation (CDA).^[^ ^55^ ^]^ With the calculated internal fields, various physical quantities such as polarization state, scattering cross section, and near‐field responses can be retrieved without expensive computations as illustrated in Figure 5b. The two‐stage method has been applied to the prediction of various responses of silicon cuboidal and plasmonic nanoantennas. Statistical results show the average errors of the far‐field prediction are well below 10% and the rate of failed prediction is of the order of 5%. It is noteworthy that the skipped connections in the U‐Net guarantee the physical information strongly related to the spatial information can be precisely inferred. Such a unique property of the two‐step approximation enhances the interpretability of the machine learning model in the application of general physical problems.

## 4 Generative Models and Dimensionality Reduction

Discriminative models are able to approximate forward simulation with extraordinary accuracy so as to enable various optimizations of the parameters defining the photonic structures and materials. However, when the dimension of the design space grows to thousands and more, it is infeasible to generate sufficient data for the training of a surrogate model. In addition, optimization is more likely to converge to local minima due to the degeneracy problem and the sparsity of the solution. Generative models, in this situation, are a candidate to reduce the dimensionality of the design space to assist the fast‐global optimization. Here, we will focus on the deep generative models, including GANs^[^ ^31^ ^]^ and VAEs,^[^ ^32^ ^]^ and their variations in the design of photonic media.

### 4.1 GANs

**Figure 6:**
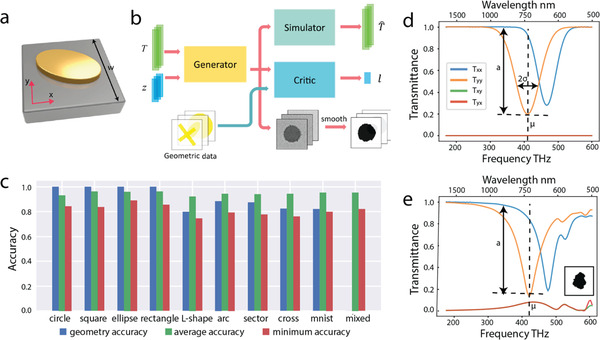
GAN‐based design of metasurface nanostructures. a) Sketch of the configuration of the metasurface unit cell. b) Architecture of the proposed network for GAN‐based photonic design. Three separate networks, a generator, a critic, and a simulator, constitute design network. c) Accuracy of the test results when different classes of geometry data are used as the training data of the GAN. d,e) Example of inverse design of metasurfaces with human‐defined spectra. d) Desired transmittance spectra as the input to the generator, where *T_xx_* and *T_yy_* are two randomly generated Gaussian‐like responses, while *T_xy_* and *T_yx_* are 0 throughout the frequency range of interest. e) The resultant unit cell generated by the model to fit the target spectra, along with the simulated transmittance spectra of this generated metasurface. Reproduced with permission.^[^ ^56^ ^]^ Copyright 2018, American Chemical Society.

As an initial attempt, a network model incorporating a GAN has been proposed to identify patterns with arbitrary topology, given desired input responses.^[^ ^56^ ^]^ The network architecture, comprised of a generator, a discriminator, and a simulator, is illustrated in **Figure** **6**a. The discriminator receives the patterns from the geometric dataset and the ones produced from the generator, guiding the generator to produce the patterns are similar to the dataset. In the meantime, the pretrained simulator enforces the generator to create the metasurface nanostructures with desired optical responses. Given certain design objectives and a geometric dataset that represents the topologies of metasurface nanostructures, the framework identifies the optimal topology from the dataset within 10 min on a GPU machine. The test accuracy of the design with different classes of geometry data are calculated and presented in Figure 6c, where the geometric accuracy reflects the possibility that the designed pattern belongs to the same class as the input dataset and the average/minimum accuracy measures the similarity of the spectra of input and generated structures. Figure 6d,e provides an example of designing a metallic metasurface in the visible and near infrared regime with a user‐defined spectrum. Figure 6d shows the desired amplitude transmittances that *T_xx_* and *T_yy_* are two random Gaussian‐like resonances and *T_xy_* and *T_yx_* are zero. The generated pattern along with its simulated response is shown in the Figure 6e. Although there exists no exact solution to spectral demand described above, the network eventually generates patterns whose spectra share common features with the input spectra including the resonance frequency, the spectral bandwidth, and the transmission magnitude.

With the excellent ability of globally exploring of the design space with reduced dimensionality, GANs have also been applied to the design of various types of photonic devices for different applications. For example, it has been proven that GANs are able to model the bidirectional mapping of optical structures and their responses for the efficient design of metallic meta‐atoms.^[^ ^57^ ^]^ An et al. proposed a GAN‐based framework for the automatic design of multifunctional dielectric metasurfaces, reducing the tremendous labor of designing large area metasurfaces for the arbitrary manipulation of phase, amplitude, and polarization of light.^[^ ^58^ ^]^ Except for metasurfaces functioning in optical frequencies, advanced GANs are implemented for the discovery of radio frequency (RF) metasurfaces.^[^ ^59^ ^]^ The designed RF metasurfaces have great potential for manipulating reflection/transmission and beam scanning/focusing in an extremely low‐profile. Recently, it has also been reported that GANs can be utilized for the design of photonic devices in optical communication such as power splitters,^[^ ^60^ ^]^ integrated photonic devices,^[^ ^61^ ^]^ and an optical invisible cloaking.^[^ ^62^ ^]^

### 4.2 VAEs

**Figure 7:**
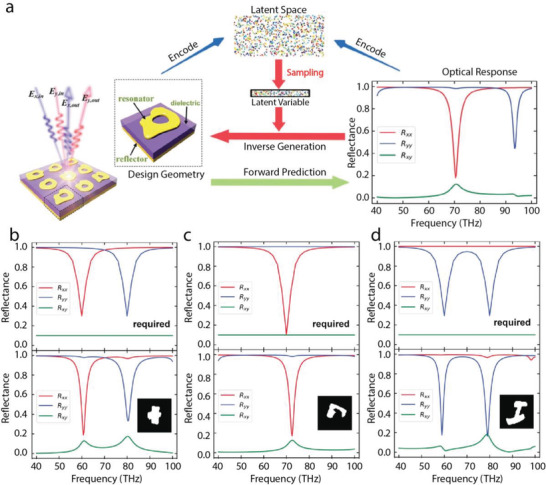
VAE‐based strategy for the photonic structure design. a) The illustration of the VAE network for the design and characterization of reflective metamaterials. A set of meta‐atoms and their optical responses are encoded into a latent space, from which the latent variables are sampled for the inverse design in response to certain objective. b–d) Required on‐demand reflection spectra input to the model. e–g) Unit cells of the VAE‐designed meta‐atoms (black patterns) and the optical responses of their corresponding metamaterials, with the input as shown in (b–d), respectively. Reproduced with permission.^[^ ^63^ ^]^ Copyright 2019, Wiley‐VCH.

As another essential member of deep generative models, VAEs have been implemented to reduce the dimensionality of photonic nanostructures and their corresponding physical properties for efficient optical design. Ma et al. utilized VAEs to encode the meta‐atoms of metamaterials and their optical responses, enabling the investigation of the complex structure‐performance relationship without extensive data collection.^[^ ^63^ ^]^ **Figure** **7** illustrates the deep generative model for metamaterial design and characterization. The metamaterial and its optical responses are encoded into the same latent space so that similar designs and optical responses are automatically clustered together. Candidate designs can be generated by sampling the latent space given requirements in the decoding process. Figure 7b–g presents three examples of on‐demand designs of metamaterials through the proposed generative model. The desired reflection spectra ranging from 40 THz to 100 THz are displayed in Figure 7b–d, and the retrieved metamaterial designs together with the simulated spectra are presented in Figure 7e–g, respectively. The spectra of the designs exactly replicate the objective. Similar techniques have also been applied to the design of multi‐layered chiral metamaterials that satisfies various chiroptical response requirements.

### 4.3 Other Approaches for Dimensionality Reduction

It is noteworthy that the reduction of dimensionality with a continuity property in the encoded space can also be fulfilled without GANs and VAEs. For example, an autoencoder (AE) and principal component analysis have been applied to the analysis of photonic dataset and revealed the underlying physical connections between structural parameters and their optical responses.^[^ ^64^ ^]^ Indeed, dimensionality reduction can also be carried out through pure mathematical tools such as Fourier transforms and wavelet transforms, which have been widely implemented to find a compact sparse representation of the parameter space in time series analysis and image processing.

Another effective way to represent the topology of a photonic structure is through a level set. Intuitively, a level set is the intersection of a 3D surface and a 2D plane. Thus, a compact representation of a 2D structure can be found by looking for the corresponding 3D surface. Usually, the level set is widely used in topology optimization methods.^[^ ^65^ ^]^ Recent research shows that level set and Fourier transforms can be combined to encode 2D photonic structures to a sparse representation.^[^ ^66^ ^]^ It is also reported that the 3D surface in the level set method can be represented by multiple control points at different heights.^[^ ^67^ ^]^ Such representation can produce an extensive range of nearly arbitrary 2D photonic structure shapes with only a few variables.

## 5 Optimization with Deep Learning Models

Machine learning as a stand‐alone technique is able to analyze high dimensional complex datasets to capture the essential features of the dataset for the approximation of the physical responses and the design of photonic structures. On the other hand, traditional optimization algorithms, such as adjoint methods, genetic algorithms, and particle swarm optimization, also have significant successes in the optimization of photonic structures and devices. However, some drawbacks of these traditional optimization algorithms prevent their use as effective approaches for the design of high DOF devices in a fast‐global manner. For example, adjoint methods calculate the gradient of the device parameters with respect to the design objective and update the parameters by subtracting the gradients. Since the gradients are computed based on the physical process formulated by the Maxwell's equations, the optimization is guaranteed to seek a local minimum. Yet, as all other gradient‐based optimization, global minimum is unlikely to be achieved if the objective cannot be formulated as a convex function.^[^ ^68^ ^]^ Stochastic optimization, such as random search and evolutionary algorithms are more likely to identify global minimum, but extensive evaluation of the performance of these designs is inevitable due to a need to explore the whole solution space. Thus, stochastic optimization algorithms are not suitable for designs that require expensive simulation to validate their performance. In order to augment the capability of these traditional optimization algorithms, we may seek help from data‐driven methods. There have been reports that coupling statistical learning and evolutionary algorithms can address the global optimization of dielectric photonic structures.^[^ ^69^ ^]^ In this section, we will focus on a few examples that incorporate deep learning models with traditional optimization algorithms to alleviate the challenges of designing high DOF photonic devices.

### 5.1 Adjoint Methods

**Figure 8:**
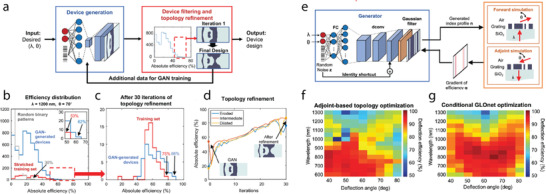
Consolidation of adjoint methods and deep learning for the design of metagratings. a–d) Design of freeform metagratings with a GAN. a) Schematic of the design strategy. Metagratings candidates are generated from a GAN with the desired input of wavelength and deflection angle. The output metastructures are further refined with topology optimization. b,c) Histograms of performances of generated metagratings compared to the topology‐optimized structures (training dataset) d) Efficiency of the GAN‐generated metagrating with additional iterations of topology optimization. The efficiency and robustness of the generated structures can be consistently improved through additional optimizations. e–g) Conditional GLOnet for global metagrating design. e) The schematic of the GLOnet, where the weights of the generator network are updated with the gradients calculated by the adjoint method. f,g) Optimized performance of the designs retrieved from the adjoint optimization and the GLOnet, respectively, given a range of desired deflection angles and wavelengths. a–d) Reproduced with permission.^[^ ^70^ ^]^Copyright 2019, Americal Chemical Society. f–h)Reproduced with permission.^[^ ^72^ ^]^ Copyright 2019, American Chemical Society.

One of the approaches to avoid the local minimum in the gradient‐based methods is to utilize deep generative models to produce vast number of new designs based on sufficiently good designs, and to optimize the generated designs to yield outperformed structures. For example, Jiang et al. proposed a joint optimization framework with GAN and adjoint methods to optimize topology‐complex 2D metagratings with high efficiency over a broad range of deflection angles and wavelengths.^[^ ^70^ ^]^ As shown in **Figure** **8**a, a GAN is trained with images of periodic, topology‐optimized metagratings. Since a GAN can capture the geometric features of the training data and produce more similar, but not identical, data, it is likely that the GAN‐generated designs jump out of local minima and present higher efficiency. Adjoint optimization can be further applied to the designs to optimize the devices with enhanced robustness and efficiencies. Iterative refining of the GAN with these new designs consistently boosts the fidelity of the design. Figure 8b shows an example of the metagrating generation and refinement process with a GAN and topology optimization. The histogram of training data, GAN‐generated metagratings, and random binary patterns are shown in Figure 8b,c. With additional topology refinement, the device efficiency and robustness are further improved as shown in Figure 8d.

Besides GANs, another generative model, adversarial autoencoders (AAEs) are also adopted for the design of high‐efficiency thermal emitters.^[^ ^71^ ^]^ An AAE is a generative architecture that incorporates an autoencoder into an adversarial learning framework and can generate more photo‐realistic images compared to vanilla GANs. Refining the AAE‐generated patterns with topological optimization has been proven to yield more robust and efficient designs as compared to the optimization solely with the adjoint method. As an example, the reported emitter with an optimized topology has a normalized efficiency of 97.9%, approaching the ideal emitter, and far exceeding the simple cylindrical emitter with an efficiency of 83%.

Global optimization with adjoint methods can also be realized by coupling the adjoint method with a generative model.^[^ ^72^ ^]^ Figure 8e presents a proposed global optimization based on a generative neural network (GLOnet). The network takes device parameters, such as wavelength and deflection angles, and a noise vector as input, and generates binary vectors representing 1D metagratings. In order to train the network so as to produce optimal devices, efficiency gradients are calculated for each device using forward and adjoint electromagnetic simulations. These gradients are backpropagated through the network to update the weights of the neurons. It is proven that the devices parametrized by a neural network with randomness at the input can almost surely converge to global minima. Figure 8f,g presents the performance comparison of adjoint methods and the GLOnet optimization. The devices designed by GLOnet in general have higher efficiencies than the devices optimized by adjoint method, while requiring lower computational cost.

### 5.2 Black‐Box Optimizations

Black‐box optimization algorithms, such as simulated annealing, random search, and evolutionary algorithms, on the other hand, do not seek the gradients to iteratively update the parameters. Instead, they stochastically update the structural parameters either through probabilistic methods or by emulating physical/biological processes to identify a solution in a global manner. However, without the gradient information from the physical model of the problem, vast iterations of computation are required to explore the space in order to yield an optimal solution. The redundant computations limit these black‐box optimizations in the design of photonic structures, materials, and devices requiring computationally intensive simulations. To alleviate the repeated computation, Hegde proposed a strategy that pairs the evolutionary algorithm with a deep neural network.^[^ ^73^ ^]^ The network model is trained as a surrogate model to partially replace the expensive simulation for the preselection and optimization of optical thin‐film systems. The massive parallelized simulation enabled by the surrogate model significantly reduces the time for evaluating the fitness/cost function during the optimization. Essentially, the network model is equivalent to a cache that stores the calculated results, but with a dramatically compressed size, for fast evaluation of the physical responses of photonic devices without repeated computations. The interpolation property of the machine learning model also expands the capacity of the cache for predicting the responses of inputs that are not exact to the stored data. Such a surrogate model is also reported in the design of various photonic structures, such as integrated photonics.^[^ ^74^ ^]^

On the other hand, the efficacy of the black‐box optimizations is also dependent on the dimensionality of the solution space, i.e., DOF of the photonic systems. Large dimensionality of the optimization space reduces the possibility of identifying a global solution. To bring down the DOF, generative models can be incorporated into the optimizations serving as a method of dimensionality reduction. As we discussed in sections 4.1 and 4.2, with sufficient empirical photonic data, GANs, VAEs, and other machine learning algorithms can construct a compact sparse representation of photonic structures in a latent space with a reduced dimensionality. Searching the latent space of the generative models is much more efficient compared to performing optimization on the original high‐dimensional data space.

**Figure 9:**
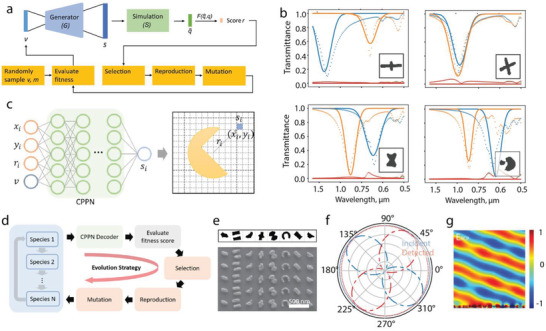
Consolidation of evolutionary algorithms and deep learning for the design of meta‐atoms. a) Flowchart of the ES‐based optimization with a generative model in the loop. The generator is responsible to recover the low‐dimensional representation of the photonic structures into the photonic design. b) Samples of on‐demand inverse design of metasurfaces. The desired spectra *T_xx_*, *T_yy_*, *T_xy_*, and *T_yx_* are shown in solid curves, while the simulated performance of the designs is shown in dashed lines. The generated patterns in the unit cell are depicted in the lower right corner of each plot. c) The schematic of a compositional pattern‐producing network (CPPN). The CPPN as a generator composes the pattern one pixel at a time. d) The flowchart of the cooperative coevolution (CC) algorithm for the design of multiple meta‐atoms in the metasurfaces. e) Designed gradient metasurface for polarization conversion and beam steering. The metasurface is able to convert portion of left circularly polarized (LCP) incidence into its counterpart (RCP) and deflect it away. f) Measured polarization states (dashed lines) of the incident and diffracted lights. The dashed‐dotted lines represent measured data after a quarter waveplate. The rotation of measured polarization with the waveplate confirms the light polarization is flipped from LCP to RCP. g) Simulated electric field distribution of RCP component under the LCP incidence. a,b) Reproduced with permission.^[^ ^75^ ^]^Copyright 2020, IEEE. c–g) Reproduced with permission.^[^ ^77^ ^]^ Copyright 2019, Wiley‐VCH.

**Figure** **9**a presents the flowchart of a strategy that consolidates generative models and an evolution strategy (ES) for photonic structure design.^[^ ^75^, ^76^ ^]^ A pretrained generator, trained either from a GAN or a VAE, is incorporated to generate photonic structures from dimensionality‐reduced latent vectors. The strategy begins with generating a population of random latent vectors, and these vectors are recovered to photonic structures for subsequent simulation. After evaluating the fitness score, the latent vectors go through selection, reproduction, and mutation, as the traditional ES algorithm. This process iterates till an optimal solution is reached. Figure 9b shows four examples of the designed photonic structures given objective transmittance *T_xx_*, *T_yy_*, *T_xy_*, and *T_yx_* at different incident polarizations.

With more sophisticated evolutionary algorithms and generative networks, the maximum DOF of the design can be further enhanced. Figure 9c–g show an example of the consolidation of cooperative coevolution (CC) and a generative model enabling the fast design of meta‐molecules composed of multiple distinct meta‐atoms.^[^ ^77^ ^]^ The generator in the example optimization framework is implemented by compositional pattern‐producing networks (CPPNs).^[^ ^78^ ^]^ Figure 9c illustrates the architecture of a CPPN. The input is a combination of a latent vector and the coordinates of an image, and the network composes the topology of photonic structure with each pixel one at a time. Such a generation process guarantees that structures with highly complex features as in training dataset can be produced. Consolidated with a cooperative CC^[^ ^79^ ^]^ framework and an ES algorithm as outlined in Figure 9d, the hybrid framework assists the design of metasurfaces with multiple meta‐atoms collaboratively manipulating a range of far‐field properties of light. Figure 9e presents a designed metasurface that is able to convert left circularly polarized (LCP) incident light to its counterpart right circularly polarized (RCP) with a phase gradient and deflects the converted portion away from the incident direction. The measured polarizations states and full wave simulation of the electric field are shown in Figure 9f,g.

## 6 Discussion and Outlook

Over the past few years, machine learning has successfully demonstrated its potential to yield complex high‐performance photonic designs with little human intervention. However, an accurate model inevitably requires a huge amount of training data, which may incur a substantial computational burden. The tradeoff between the size of the dataset and the accuracy of the model is indeed a crucial factor to be considered when machine learning is utilized for inverse design. There are several approaches being investigated to mitigate the dependency on data. For example, using advanced machine learning techniques in combination with physical methods can increase the explainability of the model and thus improve the prediction accuracy with less data.^[^ ^54^ ^]^ Meanwhile, deep learning has proved to be able to capture the fundamental law behind the complex physical phenomena,^[^ ^80^ ^]^ and serve as the intermediate steps for solving numerical partial differential equations.^[^ ^81^ ^]^ Such strategies provide a fast, generalized, and accurate modeling method for potential optimization and inverse problems with much lower data requirement. On the other hand, since collecting data does not conflict with traditional optimization strategies, data generation and optimization can be performed in parallel. The collected data can be used to train a machine learning model for the acceleration of the simulation in the later optimization steps. Lastly, as other data‐driven research, an effective approach to accelerate the development of machine‐learning‐assisted photonic design is to collectively construct large datasets of various optical designs with the effort of the optical community.^[^ ^82^ ^]^ The established dataset could avoid repeated efforts of generating simulation data, shorten the cycle of implementing new algorithms, and provide a unified standard to evaluate model performance.

The prosperity of machine learning and artificial intelligence (AI) is bringing the scientific community onto a new stage. In this era, the analytical methods, optimization algorithms, and data‐driven approaches are consolidated, forming a toolbox to uncover the theory behind complex phenomena and design unconventional devices that could never be discovered before. In the realm of optics and photonics, we have witnessed the evolution of research methodologies from analytically solving governing equations to today's AI strategies with learning and optimization. In our report, we have covered a couple of outstanding works in the emerging stages of the field. Machine learning algorithms have helped unearth the intrinsic relations between matter and light behavior, providing insights to enable and assist the design of optical components. Notwithstanding the fact that these are preliminary research, we can expect, when state‐of‐the‐art machine learning algorithms are progressively adapted to the optical community, the complexity of the design to continue growing, and the performance to further approach the limit bounded by physics. Nowadays, due to the demanding geometric and physical restrictions, the design of optical components in modern applications has heavily relied on computation and optimization algorithms. With the union of AI and machine learning, we are anticipating machine intelligence, jointly with traditional methods, to substantially boost the discovery and development of advanced photonic devices in essential and/or unconventional applications, including optical communications, high‐resolution displays, virtual/augmented reality, various sensing technologies, and so much more.

## Conflict of Interest

The authors declare no conflict of interest.
